# Continuous theta burst stimulation over the bilateral supplementary motor area in obsessive-compulsive disorder treatment: A clinical randomized single-blind sham-controlled trial

**DOI:** 10.1192/j.eurpsy.2022.2323

**Published:** 2022-10-07

**Authors:** Qihui Guo, Kaifeng Wang, Huiqin Han, Puyu Li, Jiayue Cheng, Junjuan Zhu, Zhen Wang, Qing Fan

**Affiliations:** 1 Shanghai Mental Health Center, Shanghai Jiao Tong University School of Medicine, Shanghai, China; 2 Shanghai Key Laboratory of Psychotic Disorders, Shanghai, China

**Keywords:** Bilateral supplementary motor area, continuous theta burst stimulation, obsessive-compulsive disorder, treatment

## Abstract

**Background:**

Obsessive-compulsive disorder (OCD) can cause substantial damage to quality of life. Continuous theta burst stimulation (cTBS) is a promising treatment for OCD patients with the advantages of safety and noninvasiveness.

**Objective:**

The present study aimed to evaluate the treatment efficacy of cTBS over the bilateral supplementary motor area (SMA) for OCD patients with a single-blind, sham-controlled design.

**Methods:**

Fifty-four OCD patients were randomized to receive active or sham cTBS treatment over the bilateral SMA for 4 weeks (five sessions per week, 20 sessions in total). Patients were assessed at baseline (week 0), the end of treatment (week 4), and follow-up (week 8). Clinical scales included the YBOCS, HAMD_24_, HAMA_14_, and OBQ_44_. Three behavioral tests were also conducted to explore the effect of cTBS on response inhibition and decision-making in OCD patients.

**Results:**

The treatment response rates were not significantly different between the two groups at week 4 (active: 23.1% vs. sham: 16.7%, *p* = 0.571) and week 8 (active: 26.9% vs. sham: 16.7%, *p* = 0.382). Depression and anxiety improvements were significantly different between the two groups at week 4 (HAMD_24_: *F* = 4.644, *p* = 0.037; HAMA_14_: *F* = 5.219, *p* = 0.028). There was no significant difference between the two groups in the performance of three behavioral tests. The treatment satisfaction and dropout rates were not significantly different between the two groups.

**Conclusions:**

The treatment of cTBS over the bilateral SMA was safe and tolerable, and it could significantly improve the depression and anxiety of OCD patients but was not enough to improve OCD symptoms in this study.

## Introduction

Obsessive-compulsive disorder (OCD) is a mental disorder characterized by uncontrollable, intrusive recurring obsessive thoughts and compulsive behaviors. OCD usually occurs at an early age and has a long course, which causes substantial damage to the patients’ social function and quality of life [1]. According to a follow-up study, only 6% of patients achieved full remission in a 2-year time frame [2]. OCD has been rated as one of the top 10 disabling diseases by the World Health Organization [3].

With the development of neuroimaging technology, the neural mechanism of OCD has been discovered gradually. The cortico-striato-thalamo-cortical circuits have received the most evidence-based support. In this neural network, the prefrontal cortex (PFC) (containing the orbito-frontal cortex [OFC], medial PFC [mPFC], and anterior cingulate cortex [ACC]), supplementary motor area (SMA) and premotor areas, striatum, globus pallidus, and thalamus show hyperexcitability in OCD patients [4–7].

Cognitive abnormalities were also found in patients with OCD, including defects in memory, attention, and executive function [8, 9]. OCD patients have negative attention biases and easily notice threatening information. Response inhibition (RI), one type of executive function, is considered to be the core defect of OCD and is linked to the symptom “cannot stop thinking.” RI means the ability to consciously inhibit undesirable and inappropriate thoughts and behaviors [10, 11]. Therefore, RI can be divided into cognitive inhibition and behavioral inhibition. The Stroop task is usually used to measure cognitive inhibition, and the Go/No-go task or stop-signal task (SST) are used to measure behavioral inhibition. Compared with healthy control subjects, patients with OCD show more errors and longer reaction times in the Stroop task and have more errors in the Go/No-go task [12–14]. It has been confirmed that RI is correlated with the frontobasal ganglia circuit, and the pre-SMA is one of the relevant brain regions [15, 16].

Pharmacotherapy and psychotherapy are the first-line treatments for OCD. Selective serotonin reuptake inhibitors (SSRIs) are widely used in clinical treatment and have been proved to be more effective than placebos [17, 18]. Cognitive behavior therapy (CBT) combined with response prevention (ERP) is also a good treatment for OCD [19, 20]. However, approximately 40% of patients have no response to these two therapies [21]. The curative efficacy of pharmacotherapy and psychotherapy is still not satisfactory; their efficacy rates range from 40 to 60% [22], and over 60% of patients will relapse [23]. Moreover, for patients who have no response to SSRIs and CBT, an increased risk of adverse events is seen in the long term [3]. Therefore, it is meaningful to develop a new treatment strategy.

Progress has been continuously made in neural regulation for treating OCD. In particular, transcranial magnetic stimulation (TMS) as a safe and noninvasive treatment has a great advantage [24]. By creating a fast-changing magnetic field, TMS can modulate neural excitability, provoking the hyperpolarization or depolarization of surface cortical neurons [25]. TMS can be divided into single-pulse TMS, paired-pulse TMS, repetitive TMS (rTMS), and theta burst stimulation (TBS) [24]. Among these, rTMS and TBS are used for treatment. Research has shown that high-frequency (≥5 Hz) stimulation can enhance neural excitability [26], and low-frequency (≤1 Hz) stimulation can inhibit neural excitability [27]. rTMS uses a repeated fixed-frequency single pulse to stimulate the cerebral cortex, while TBS embeds three burst high-frequency theta stimulations (50 Hz) into a 5 Hz persistent stimulation with an interval of 200 ms [28]. Compared with rTMS, TBS is closer to the biological rhythm and can improve the induction of synaptic long-term potentiation [29, 30]. TBS can be further divided into continuous theta burst stimulation (cTBS) and intermittent theta burst stimulation (iTBS). The former has an inhibitory effect, while the latter has an active effect on the cerebral cortex.

In recent years, rTMS has showed a beneficial effect on OCD [31]. The mPFC, OFC, and SMA are the three main stimulation targets. According to two meta-analyses, high frequency (HF) stimulation over the mPFC could improve OCD symptoms [31, 32]. The efficacy of dTMS (HF) and iTBS over the mPFC has also been shown [33–35]. Therefore, the mPFC is not an ideal target for cTBS, which is a type of low-frequency stimulation. Moreover, the OFC is a challenging target, as the location of stimulation is close to the brow bones. According to Rehn et al.’s [36] meta-analysis, SMA is a promising rTMS target for OCD. Therefore, SMA was selected as the target in this study.

To explore the treatment efficacy of cTBS over the bilateral SMA in patients with OCD, this study developed a randomized sham-controlled trial, which included a 4-week treatment period and a 4-week follow-up period.

## Methods

### Study design

This study was a clinical, randomized, single-blind sham-controlled trial conducted in Shanghai Mental Health Center with active enrollment from 2019 to 2021. The study was approved by the local ethics institutional review board (IORG0002202) and registered at http://chictr.org.cn (ChiCTR1900026020). All of the patients signed informed consent forms.

### Participants

**Figure 1.**
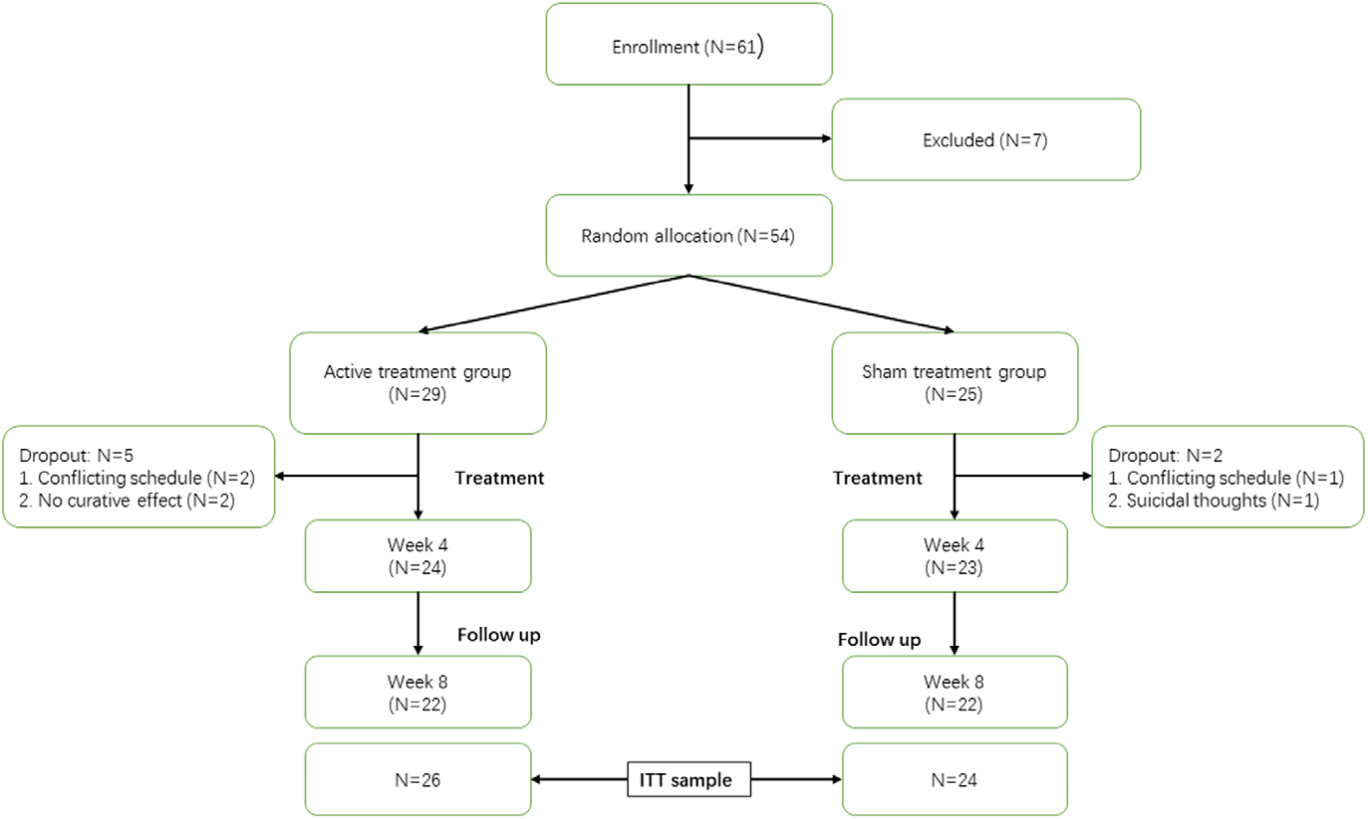
CONSORT diagram. Week 4 represents the end of cTBS treatment; week 8 represents the end of follow-up; ITT, intention to treat.

OCD patients (*N* = 61) were recruited in Shanghai Mental Health Centre through recommendations of psychiatrists and posters placed in hospital hall. Ultimately, 54 patients met the inclusion and exclusion criteria (Figure 1).

### Inclusion criteria

All patients were treated for obsessive and compulsive symptoms and were diagnosed by psychiatrists to ensure that they met the OCD criteria of the DSM-5. Age was limited to 18 ~ 54 years old, and education level was limited to above middle school. Patients must have a score between 16 and 31 on the Yale–Brown obsessive-compulsive scale (16 ≤ YBOCS ≤ 31), because patients with severe OCD symptoms could not complete the study.

### Exclusion criteria

Patients were assessed by the MINI (International Neuropsychiatric Interview) to eliminate patients with any psychiatric comorbid disorders. Patients who had a high risk of suicide or had severe physiological diseases and neurological disorders were excluded. Moreover, drug abusers and patients with ear diseases or those who used hearing aids were not allowed to participate in the research. Those who had received TMS treatment but had no significant effectiveness were also excluded.

### Treatment

Participants were divided into two groups: the active (cTBS) group and the sham (control) group. The active group received cTBS for a month. There were five cTBS treatments a week (from Monday to Friday), and 20 cTBS treatments in total. Treatment was administered by a Ma GPro X100 device (Maga Venture) with a Cool-MCF-B65 butterfly coil. According to the 10/20 International EEG Positioning System, the coil was placed anterior to the apex of the skull and located 15% of the distance between the nasion and the occipital eminence. The coil was 45° from the SMA, and treatment was performed on one side and then the other side instead of treating the bilateral SMA at the same time.

The resting motor threshold (RMT) was quantified as the minimum stimulus intensity at which 5/10 of the single pulse trials to the contralateral primary motor cortex elicited a threshold electromyogram response (50 mV in the peak-to-peak amplitude) in the contralateral abductor pollicis brevis. The detailed parameters of cTBS were as follows: the intensity of stimulus was 110% RMT; the frequency of trains was 50 Hz and the number of pulses was three; and the frequency of intertrain intervals was 5 Hz and pulses number was 200. Every treatment contained 1,200 pulses and lasted for 48 s. The average stimulator intensity was 40% RMT in both groups.

The treatment of the sham group was the same as active group. The only difference was that the coil was flipped 180° in the sham group. The device also made the same sound but could not stimulate the brain. The medication regimen of the participants at baseline was maintained throughout the cTBS treatment and follow-up periods.

### Assessment

The reduction rate of the YBOCS score was used as the primary outcome. Hamilton depression scale (HAMD_24_) and Hamilton anxiety scale (HAMA_14_) scores were used as secondary outcomes. A ≥ 35% reduction in YBOCS score was defined as “treatment response,” also known as “full response,” and a ≥ 25% reduction in YBOCS score was defined as “partial response” [37].

The OBQ_44_ (Obsessive Belief Questionnaire, OBQ_44_) was used to measure the severity of obsessive belief, which was one of the exploratory outcomes in this study. Moreover, behavioral tests, including the Stroop task, SST and probabilistic reasoning task (PRT), were also regarded as exploratory outcomes. The Stroop task and SST were used to explore the effect of cTBS on RI. PRT, a paradigm which simulates the process of decision-making during evidence accumulation [38, 39], was used to explore the effect of cTBS on decision-making.

**Figure 2.**
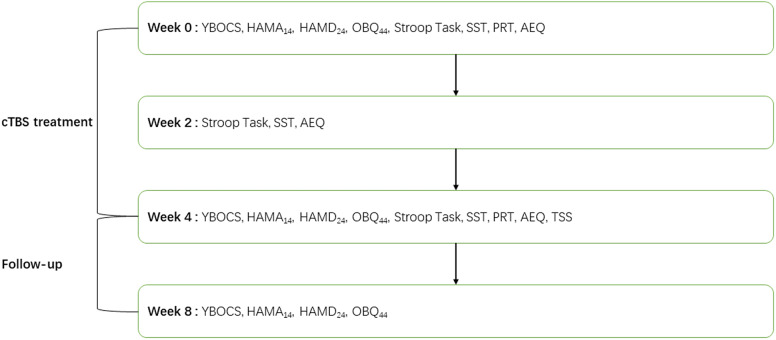
Experimental workflow. AEQ, Adverse events questionnaire; HAMD_24_, Hamilton depression scale; HAMA_14_, Hamilton anxiety scale; OBQ_44_, obsessive belief questionnaire; PRT, probabilistic reasoning task; SST, stop-signal task; TSS, treatment satisfaction scale; YBOCS, Yale-Brown obsessive-compulsive disorder scale.

We developed the treatment satisfaction scale (TSS) to evaluate patients’ satisfaction with the treatment. The adverse events questionnaire (AEQ) was developed to investigate adverse events during 1 month of cTBS treatment. The TSS score, AEQ score, and dropout rate were included as important parameters to evaluate the safety of cTBS treatment. For a detailed experimental workflow, please see Figure 2.

### Blinding

At first, patients were randomly assigned to the active or sham group through a random series number generated by a computer. As both groups received the same treatment process, the patients were blinded to the treatment conditions. Moreover, assessors were also blinded to the treatment conditions. Every patient was assigned a unique number, and only the experimenters and cTBS operators knew the detailed information.

### Statistical analysis

To ensure the reliability of result, the intention-to-treat (ITT) analysis set includes all participants who had received at least one active or sham treatment, using last observation carried forward to fill missing values.

All statistical analyses were worked on SPSS, version 24.0 (IBM Corp., Armonk, NY). Both independent T-test (two-tailed) and Pearson χ^2^ test were used to compare the difference of demographic variables and clinical features at baseline between the active group and sham group. Response rate of outcomes and satisfaction for treatment between the two groups were compared by independent T-test (two-tailed) or Fisher exact test for effective differences. Two-ways repeated measures ANOVA was used to analyze the main effect and interactive effect of Group (active group, sham group) and Time (week 0, 4, 8). Mauchly sphericity test was essential to make sure the data satisfy the sphericity hypothesis before the two-ways repeated measures ANOVA. If the data not satisfied the sphericity hypothesis, the Greenhouse–Geisser adjustment was needed. The confidence interval was set as 95%.

## Results

Twenty-four patients in active group completed cTBS treatment and 23 patients in the sham group completed sham cTBS treatment. Up to week 8, both the active group and sham group had 22 patients who completed the follow-up (Figure 1). The ITT sample included 50 patients, among whom 26 patients were included in the active group and 24 patients were included in the sham group.

### Baseline assessment

Table 1 presents the demographic and clinical features of the participants at baseline. There were no significant differences between the two groups in terms of demographic features. There was also no significant difference at baseline between the two groups on the YBOCS, HAMD_24_, HAMA_14_, and OBQ_44_ scores.

**Table 1.** Demographic data and clinical characteristic in baseline.

Characteristic	Active group (*n* = 26)	Sham group (*n* = 24)	*t*/χ2	*p*
Gender (M/F)	18/8	15/9	0.252	0.616
Age (years)	35.000 (9.520)	30.290 (7.800)	1.904	0.063
Education (years)	14.16(2.982)	13.38(3.062)	0.909	0.368
Age of onset (years)	21.32(8.479)	20.63(7.728)	0.299	0.766
Duration of illness (years)	114.81(92.601)	82.61(75.314)	1.324	0.192
YBOCS	23.690 (5.221)	24.21 (5.267)	−0.348	0.730
O-YBOCS	11.96 (2.720)	12.63 (2.584)	−0.882	0.382
C-YBOCS	11.73 (3.269)	11.58 (3.020)	0.165	0.869
HAMD_24_	15.82(7.34)	17.21 (8.29)	−0.625	0.535
HAMA_14_	13.30 (7.02)	14.25 (7.25)	−0.471	0.640
OBQ_44_	184.079 (44.879)	189.043 (38.780)	−0.417	0.679

Abbreviations: C-YBOCS, compulsion sub-score of Yale–Brown obsessive-compulsive scale; HAMD_24_, Hamilton depression scale; HAMA_14_, Hamilton anxiety scale; OBQ_44_, obsessive belief questionnaire; O-YBOCS, obsession sub-score of Yale–Brown obsessive-compulsive scale; YBOCS, Yale-Brown obsessive-compulsive disorder scale.

### Primary outcomes

All the interactions of Time × Group on the YBOCS, O-YBOCS (obsession sub-score of the Yale–Brown obsessive compulsive scale), and C-YBOCS (compulsion sub-score of the Yale–Brown obsessive compulsive scale) were not statistically significant. All the Time effects of the YBOCS, O-YBOCS, and C-YBOCS were statistically significant. For detailed results, please see Table 2.

**Table 2.** Effect of cTBS treatment on primary and secondary outcomes.

Outcome			End of treatment (RANOVA)				End of follow-up (RANOVA)			
	Mean (SD)		Time Effect		Time × Group interaction		Time effect		Time × Group interaction	
	Active (*N* = 26)	Sham (*N* = 24)	F	p	F	p	F	p	F	p
*YBOCS*			55.150	0.000	1.100	0.300	43.65^a^6	0.000	0.683^a^	0.466
Week 0	23.690 (5.221)	24.21 (5.267)								
Week 4	18.654 (6.279)	20.417 (6.426)								
Week 8	18.615 (6.487)	20.083 (6.520)								
*O-YBOCS*			39.994	0.000	0.215	0.645	35.485^a^	0.000	0.131^a^	0.844
Week 0	11.962 (2.720)	12.625 (2.584)								
Week 4	9.500 (3.165)	10.500 (3.323)								
Week 8	9.385 (3.213)	10.208 (3.336)								
*C-YBOCS*			39.417	0.000	1.814	0.184	26.816^a^	0.000	1.103^a^	0.844
Week 0	11.731 (3.269)	11.583 (3.020)								
Week 4	9.154 (3.728)	9.917 (3.387)								
Week 8	9.231 (3.881)	9.875 (3.443)								
*HAMD*_24_			6.689	0.014	4.644	0.037	7.198	0.001	2.642	0.078
Week 0	15.667 (8.193)	17.350 (9.115)								
Week 4	9.619 (6.273)	16.800 (11.312)								
Week 8	10.095 (6.956)	14.200 (11.321)								
*HAMA*_14_			6.643	0.014	5.219	0.028	2.724	0.072	1.861	0.162
Week 0	13.191 (7.846)	14.350 (7.975)								
Week 4	7.381 (5.005)	14.000 (9.476)								
Week 8	9.333 (7.296)	13.050 (11.546)								
*OBQ*_44_			7.221	0.010	0.752	0.390	13.437	0.000	1.136	0.314
Week 0	184.079 (44.880)	189.043 (38.780)								
Week 4	165.692 (40.858)	179.626 (50.165)								
Week 8	152.269 (38.318)	171.470 (57.644)								

*Note*: End of treatment (RANOVA), two-ways (Group × Time (week 0, 4)) repeated measures ANOVA; end of follow-up (RANOVA) = two-ways (Group × Time (week 0, 4, 8)) repeated measures ANOVA.

Abbreviations: C-YBOCS, compulsion sub-score of Yale–Brown obsessive-compulsive scale; HAMD_24_, Hamilton depression scale; HAMA_14_, Hamilton anxiety scale; OBQ_44_, obsessive belief questionnaire; O-YBOCS, obsession sub-score of Yale–Brown obsessive-compulsive scale; YBOCS, Yale-Brown obsessive-compulsive disorder scale.

a Correction for nonsphericity.

**Figure 3.**
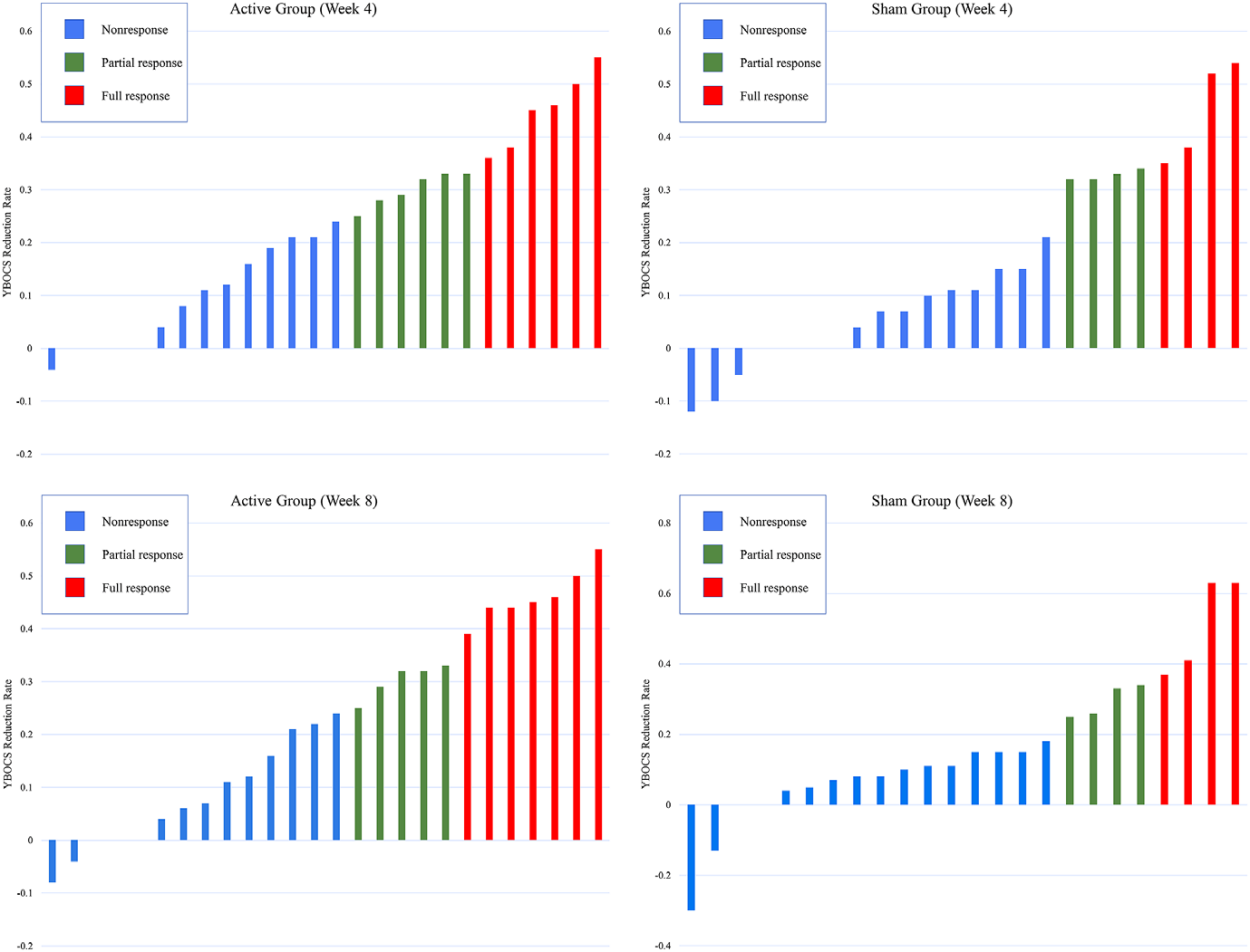
Individual distribution of full responders and nonresponders according to the reduction rate of YBOCS at week 4 and week 8. YBOCS, Yale-Brown obsessive-compulsive scale; week 4: YBOCS reduction rate = [YBCOS (week 0)−YBCOS (week 4)]/YBCOS (week 0); week 8: YBOCS reduction rate, [YBCOS (week 0)−YBCOS (week 8)]/YBCOS (week 0).

The treatment response rate (full response: a reduction ≥35% in the YBOCS score) at week 4 was 23.1% (6/26) in the active group and 16.7% (4/24) in the sham group, and there was no significant difference between the two groups (χ^2^ = 0.321, *p* = 0.571). The treatment response rate at week 8 was 26.9% (7/26) in the active group and 16.7% (4/24) in the sham group, and there was no significant difference between the two groups (χ^2^ = 0.765, *p* = 0.382). For detailed results, please see Table 3 and Figure 3.

**Table 3.** Response rate after cTBS treatment and follow-up.

YBOCS	Time	Active (%) group	Sham (%) group	χ2	*p*
Treatment Response	Week 4	23.1(6/26)	16.7(4/24)	0.321	0.571
	Week 8	26.9(7/26)	16.7(4/24)	0.765	0.382
Partial Response	Week 4	46.2(12/26)	33.3(8/24)	0.855	0.399
	Week 8	46.2(12/26)	33.3(8/24)	0.855	0.399

*Note:* Treatment response, a reduction ≥35% in the YBOCS score; partial response, a reduction ≥25% in the YBOCS score.

Abbreviation: YBOCS, Yale–Brown obsessive-compulsive scale.

### Secondary outcomes

At week 4, both HAMD_24_ and HAMA_14_ scores had significant Time effects (HAMD_24_: *F* = 6.689, *p* = 0.014; HAMA_14_: *F* = 6.643, *p* = 0.014) and Time × Group interaction (HAMD_24_: *F* = 4.644, *p* = 0.037; HAMA_14_: *F* = 5.219, *p* = 0.028). At week 8, the Time effect of the HAMD_24_ and HAMA_14_ scores was still significant (HAMD_24_: *F* = 7.198, *p* = 0.001; HAMA_14_: *F* = 2.724, *p* = 0.072), but the Time × Group interaction of the HAMD_24_ and HAMA_14_ was not significant (HAMD_24_: *F* = 2.642, *p* = 0.078; HAMA_14_: *F* = 1.861, *p* = 0.162). Whether after treatment or after follow-up, the Time effect and Time × Group interaction were not significant in OBQ_44_ scores. For detailed results, please see Table 2.

**Figure 4.**
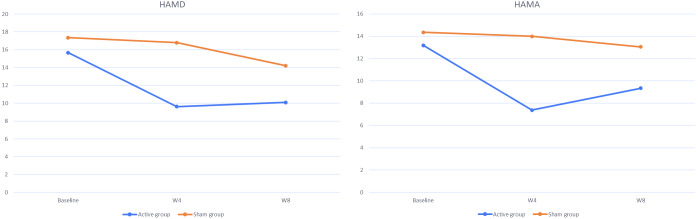
Changes of HAMD and HAMA scores between the active group and the sham group. Baseline, week 0; W4, week 4; W8, week 8.

Simple effect analysis (Table 4) indicated that the HAMD_24_ and HAMA_14_ scores of the active group between week 0 and week 4 were significantly different (HAMD_24_: *D*-value = 6.048, *p* = 0.005; HAMA_14_: *D*-value = 5.810, *p* = 0.004), but the HAMD_24_ and HAMA_14_ scores of the active group between week 4 and week 8 were not significantly different. Moreover, these differences were not significant for the sham group (Table 4). The changes of the HAMD_24_ and HAMA_14_ scores are displayed in Figure 4.

**Table 4.** Simple effect analysis of HAMD_24_ and HAMA_14_.

Scale	Group	Comparison	*D*-value	*p*
HAMD_24_	Active group	Week0–Week4	6.048	0.005
		Week0–Week8	5.571	0.009
		Week4–Week8	−0.476	0.983
	Sham group	Week0–Week4	0.550	0.987
		Week0–Week8	3.150	0.247
		Week4–Week8	2.600	1.488
HAMA_14_	Active group	Week0–Week4	5.810	0.004
		Week0–Week8	3.857	0.240
		Week4–Week8	−1.952	0.714
	Sham group	Week0–Week4	0.350	0.996
		Week0–Week8	1.300	0.919
		Week4–Week8	0.950	0.957

Abbreviations: HAMD_24_, Hamilton depression scale; HAMA_14_, Hamilton anxiety scale.

### Behavioral tests

In total, three behavioral tests were used in this study: the Stroop task, SST, and PRT. None of the parameters of the SST or PRT had statistically significant Time × Group interaction (Table 5). The Time × Group interaction of the Stroop task showed significance (*F* = 3.255, *p* = 0.045), but the results of the simple effect analysis indicated that there were no differences between any level of variables (Supplementary Table S1).

**Table 5.** Results of behavioral tests (Stroop task, stop-signal task).

Outcomes	Mean (SD)		Time effect		Time × Group interaction	
	Active	Sham	*F*	*p*	*F*	*p*
*Effect size* (Stroop)	*N* = 20	*N* = 15				
Week 0	48.86 (56.51)	86.48 (92.48)	0.311	0.734	3.255	0.045
Week 2	78.72 (84.72)	71.72 (76.49)				
Week 4	82.46 (68.81)	45.01 (69.52)				
*SSRT (SST)*	*N* = 20	*N* = 20				
Week 0	109.85 (166.63)	155.11 (85.40)	5.205^a^	0.011	0.61^a^	0.524
Week 2	189.28 (66.79)	204.56 (102.85)				
Week 4	161.50 (85.30)	163.70 (132.98)				
*RT (SST)*	*N* = 20	*N* = 20				
Week 0	566.81(91.52)	595.27 (70.95)	8.991	0.000	2.209	0.117
Week 2	632.65 (78.00)	611.59 (84.24)				
Week 4	644.10 (60.26)	625.61 (66.42)				
*SSD (SST)*	*N* = 20	*N* = 20				
Week 0	456.96 (158.85)	440.16 (108.45)	2.669^a^	0.091	0.129^a^	0.825
Week 2	443.37 (119.40)	407.03 (131.78)				
Week 4	482.61 (98.63)	461.91 (130.15)				
*RT (PRT)*	*N* = 15	*N* = 17				
Week 0	16.82 (12.64)	14.65 (5.73)	11.086	0.002	3.810	0.060
Week 4	8.39 (4.71)	12.31 (5.82)				
*SW (PRT)*	*N* = 15	*N* = 17				
Week 0	1.87 (1.06)	1.74 (0.49)	7.164	0.012	3.979	0.055
Week 4	1.18 (0.46)	1.63 (0.63)				

Abbreviations: PRT, probabilistic reasoning task; RT, reaction time; SSD, stop signal delay; SSRT, stop-signal reaction time; SST, stop-signal task; SW, sum weight.

a correction for nonsphericity.

### Satisfaction

The TSS was administered at week 4 after the patients completed the cTBS treatment. Seven degrees were used to measure satisfaction (1, very dissatisfied; 2, dissatisfied; 3, a little dissatisfied; 4, neutral; 5, a little satisfied; 6, satisfied; 7, very satisfied). Regarding the mean degrees of treatment satisfaction, the active group received 5.06 points (SD = 0.87) and the sham group received 4.82 points (SD = 0.73), with no statistically significant difference between the two groups (*t* = 1.044, *p* = 0.302).

### Adverse events and dropouts

Adverse events were reported for one patient in the active group and two patients in the sham group. One patient in the active group reported facial muscle twitching during the first two cTBS treatments. One patient in the sham group reported that his anxiety increased during the first three cTBS treatment, but gradually adapted later. One patient in the sham group reported a feeling of debility after the sixth cTBS treatment, but recovered after half an hour. The dropout rate between the two groups was not statistically significant (active group, 5/29; sham group, 2/25; χ^2^ = 0.289, *p* = 0.426).

## Discussion

The results indicate that cTBS treatment over the bilateral SMA could improve OCD symptoms, but there was no statistically significant difference in treatment efficacy between cTBS and placebo treatment. This conclusion is similar to a previous study [40]. Germaneau et al. [40] developed the first randomized controlled trial (RCT) that treated OCD patients with cTBS, and found that cTBS treatment over the pre-SMA was not sufficient to improve OCD symptoms. In their study, the cTBS treatment (600 pulses, 70% RMT) lasted for 6 weeks, with five sessions a week. The author commented that the small number of pulses and low threshold intensity caused the failure of the cTBS treatment [40]. Our study used a higher intensity (110% RMT) and a larger number of pulses (1,200 pulses) but also obtained negative results.

Treatment with cTBS over the SMA can significantly improve the anxiety and depression of OCD patients according to the simple effect analysis of HAMD_24_ and HAMA_14_ scores. Many studies support the opinion that theta burst stimulation over the left dorsolateral prefrontal cortex is an efficient treatment for major depressive disorder (MDD), which can significantly improve depression of MDD patients [41–43]. In 2021, a study found that cTBS over the OFC could significantly improve anxiety and depression in OCD patients [44]. Our results indicate that cTBS over the SMA may also be a significant way to improve anxiety and depression in OCD patients.

Performance on the Stroop task and SST showed no significant improvement after cTBS treatment. The hyperexcitability of the SMA is an important neural mechanism defect for OCD patients [45], and extensive connections have been found between the SMA and frontal lobe [46]. Execution control is the characteristic function of the frontal lobe. Based on the above evidences, we supposed that the excitability of the SMA was negatively correlated with RI. The results of the Stroop task and SST indicated that RI had no significant improvement after cTBS treatment.

cTBS using the Cool-MCF-B65 butterfly coil was considered to be well tolerated by the OCD patients for the reason that no severe adverse events occurred. And most patients evaluated the cTBS treatment as somewhat satisfactory. In general, cTBS is a safe and acceptable treatment for OCD patients, which is consistent with the conclusions of previous studies [40, 47, 48].

SMA plays an important role in the neural mechanism of OCD [49]. The first RCT of rTMS over the SMA was conducted by Mantovani et al. [50] in 2010, who found that the improvement of OCD symptoms in the active group was better than that in the sham group. This finding has attracted many OCD researchers’ attention to the SMA. In 2012 and 2016, Gomes et al. and Hawken et al. separately conducted the second and third RCTs of rTMS over the SMA. Both also found a significant difference in treatment efficacy between rTMS and placebo treatment [47, 51]. However, three RCTs separately reported in 2016, 2018, and 2019 reached a negative result: rTMS/cTBS over pre-SMA was not an effective treatment for OCD [40, 52, 53]. Comparing the above six RCT studies [40, 47, 50–53] with two different conclusions, we speculate three explanations for our negative results.

Firstly, in this study, the duration and quantity of cTBS treatment may not be sufficient. After cTBS treatment (week 4), the depression and anxiety of OCD patients in the active group were significantly improved compared with that in the sham group. However, the difference between the two groups was not maintained during the follow-up period. This finding indicates that 20 sessions of cTBS can improve the emotional symptoms (depression and anxiety) of OCD patients but is not enough to improve OCD symptoms. Although a previous study already conducted 30 sessions of cTBS treatment over the SMA still obtained negative results [40], the reference value is limited because the parameters were different from those in our study. Therefore, it is still meaningful and necessary to increase the duration and quantity of the cTBS intervention in our future study.

Secondly, the heterogeneity of the OCD patients may contribute to our negative results. Among the six RCT studies [40, 47, 50–53], there are three RCT studies (one obtained positive results and two obtained negative results) requiring the sample population must be treatment refractory OCD patients [40, 47, 52]. And Hegde et al. [54] suggested that rTMS over the pre-SMA may not be helpful in treatment refractory OCD patients. In our study, treatment refractory OCD patients were not identified in the sample, so there may have been an instability interference factor.

Thirdly, the stimulus parameters of cTBS in our study may not be appropriate. Stimulus parameters (including the frequency, intensity, and intervention time) have a great impact on treatment efficacy. In particular, the stimulus intensity influenced the response to cTBS treatment [55]. The RMT parameter is controversial, Germaneau et al. [40] adopted 70% RMT, and our study adopted 110% RMT. Further studies are needed to explore the optimal parameters.

## Strengths and Limitations

The current study updates knowledge on the efficacy of cTBS over the SMA for treating OCD. Compared to previous RCT studies looking at rTMS/cTBS over the SMA treatment for treating OCD [40, 47, 50–53], the current study has the largest sample size.

The insufficient accuracy of neuro-navigation is an important limitation. This study adopted the 10/20 International EEG Positioning System to position the bilateral SMA, instead of a more advanced neuro-navigation system based on magnetic resonance imaging. The position deviation may lead to the deviation of cTBS stimulation, which means that not every patient’s SMA received the equal treatment.

## Conclusions

The results suggested that cTBS over the bilateral SMA was a safe and tolerable treatment for OCD patients, and it could significantly improve the depression and anxiety of OCD patients, but was not enough to improve OCD symptoms in this study. Further studies are needed to improve the efficacy by increasing the duration and quantity and identifying the optimal parameters of cTBS treatments.

## Data Availability

The data that support the findings of this study are publicly available. The corresponding author can be contacted upon reasonable request.

## Supplementary material

For supplementary material accompanying this paper visit https://doi.org/10.1192/j.eurpsy.2022.2323. click here to view supplementary material
