# Continuous theta burst stimulation over the bilateral supplementary motor area in obsessive-compulsive disorder treatment: A clinical randomized single-blind sham-controlled trial – CORRIGENDUM

**DOI:** 10.1192/j.eurpsy.2025.21

**Published:** 2025-03-04

**Authors:** Qihui Guo, Kaifeng Wang, Huiqin Han, Puyu Li, Jiayue Cheng, Junjuan Zhu, Zhen Wang, Qing Fan

**Keywords:** Bilateral supplementary motor area, continuous theta burst stimulation, obsessive-compulsive disorder, treatment, corrigendum

This article contains an error in Table 1, which in one of the row headings refers to the duration of illness (years). This should be the duration of illness (months). The correct version of the table is presented below.

**Table 1.** Demographic data and clinical characteristic in baseline

Characteristic	Active group (*n* = 26)	Sham group (*n* = 24)	*t/χ*2	*p*
Gender (M/F)	18/8	15/9	0.252	0.616
Age (years)	35.000 (9.520)	30.290 (7.800)	1.904	0.063
Education (years)	14.16 (2.982)	13.38 (3.062)	0.909	0.368
Age of onset (years)	21.32 (8.479)	20.63 (7.728)	0.299	0.766
Duration of illness (months)	114.81 (92.601)	82.61 (75.314)	1.324	0.192
YBOCS	23.690 (5.221)	24.21 (5.267)	–0.348	0.730
O-YBOCS	11.96 (2.720)	12.63 (2.584)	–0.882	0.382
C-YBOCS	11.73 (3.269)	11.58 (3.020)	0.165	0.869
HAMD_24_	15.82 (7.34)	17.21 (8.29)	–0.625	0.535
HAMA_14_	13.30 (7.02)	14.25 (7.25)	–0.471	0.640
OBQ_44_	184.079 (44.879)	189.043 (38.780)	–0.417	0.679

Abbreviations: C-YBOCS, compulsion sub-score of Yale–Brown obsessive-compulsive scale; HAMD_24_, Hamilton depression scale; HAMA_14_, Hamilton anxiety scale; OBQ_44_, obsessive belief questionnaire; O-YBOCS, obsession sub-score of Yale–Brown obsessive-compulsive scale; YBOCS, Yale-Brown obsessive-compulsive disorder scale

Secondly, the Methods: Treatment section of the article also contains an error in the sentence “The average stimulator intensity was 40% RMT in both groups.” This should read “The average stimulator intensity was 40% MSO (maximal stimulator output) in both groups.”

## References

[r1] Guo Q, Wang K, Han H, Li P, Cheng J, Zhu J, Wang Z, Fan Q (2022). Continuous theta burst stimulation over the bilateral supplementary motor area in obsessive-compulsive disorder treatment: A clinical randomized single-blind sham-controlled trial. European Psychiatry, 65(1), e64, 1–10 10.1192/j.eurpsy.2022.2323 36203323 PMC9641651

