# Supplementary material for: Continuous theta burst stimulation over the bilateral supplementary motor area in obsessive-compulsive disorder treatment: A clinical randomized single-blind sham-controlled trial
Source: Eur Psychiatry. 2022 Oct 7;65(1):e64. doi: 10.1192/j.eurpsy.2022.2323 (PMC9641651; doi:10.1192/j.eurpsy.2022.2323)
Supplement: Supplementary file 1 [file S0924933822023239sup001.docx]

**Supplementary Table 1. Simple effect analysis of Stroop task.**

| Outcome | Group | Comparison | D-value | p |
| --- | --- | --- | --- | --- |
| Effect size (Stroop) | Active group | Week0-Week2 | -29.857 | 0.323 |
|  | (N=20) | Week0-Week4 | -33.601 | 0.141 |
|  |  | Week2-Week4 | -3.744 | 0.998 |
|  | Sham group | Week0-Week2 | 14.756 | 0.876 |
|  | (N=15) | Week0-Week4 | 41.472 | 0.106 |
|  |  | Week2-Week4 | 26.715 | 0.670 |
